# The potential renoprotective effect of Raloxifene in renal ischemia-reperfusion injury in a male rat model

**DOI:** 10.25122/jml-2023-0100

**Published:** 2023-08

**Authors:** Raghad Abdul Hameed Ali, Murooj Altimimi, Najah Rayish Hadi

**Affiliations:** 1 Department of Pharmacology and Therapeutics, Faculty of Medicine, University of Kufa, Kufa, Iraq

**Keywords:** potential renoprotective effect, Raloxifene, renal ischemia-reperfusion injury, IRI: Ischemia Reperfusion Injury, GFR: Glomerular Filtration Rate, AKI: Acute Kidney Injury, TECs: Tubular Epithelial Cells, ROS: Reactive Oxygen Species, TNF-α: Tumor Necrosis Factor, SERM: Selective Estrogen Receptor Modulator, IACUC: Institutional Animal Care and Use Committee, TAC: Total Antioxidant Capacity, SOD: Superoxide Dismutase, RAL: Raloxifene Hydrochloride

## Abstract

Renal ischemia-reperfusion injury is caused by a temporary reduction in oxygen-carrying blood flow to the kidney, followed by reperfusion. During ischemia, kidney tissue damage induces overproduction of reactive oxygen species, which produces oxidative stress. The blood flow restoration during the reperfusion period causes further production of reactive oxygen species that ends with apoptosis and cell death. This study aimed to investigate the potential renoprotective effects of Raloxifene on bilateral renal ischemia-reperfusion injury in rats by looking into kidney function biomarkers, urea and creatinine, inflammatory cytokines, such as tumor necrosis factor-alpha (TNF-α) and interleukin-1 beta (IL-1β). Additionally, antioxidant markers such as total antioxidant capacity (TAC) and the pro-apoptotic marker caspase-3 were assessed. Histopathological scores were also employed for evaluation. Our experimental design involved 20 rats divided into four groups: the sham group underwent median laparotomy without ischemia induction, the control group experienced bilateral renal ischemia for 30 minutes followed by 2 hours of reperfusion, the vehicle group received pretreatment with a mixture of corn oil and dimethyl sulfoxide (DMSO) before ischemia induction, and the Raloxifene-treated group was administered Raloxifene at a dose of 10 mg/kg before ischemia induction, followed by ischemia-reperfusion. Urea and creatinine, TNF-α, IL-1β, and caspase-3 in the Raloxifene group were significantly lower compared to the control and vehicle groups. On the other hand, TAC levels in the Raloxifene group were significantly higher than in the control and vehicle groups. This study concluded that Raloxifene had a renoprotective impact via multiple actions as an anti-inflammatory, anti-apoptotic, and antioxidant agent.

## INTRODUCTION

The term "ischemia-reperfusion injury" (IRI) is used to describe the structural and functional changes that occur when blood flow is restored following an ischemic period. Beyond the harmful effects of ischemia, restoring blood flow can have potentially harmful consequences like irreversibly damaged cell necrosis, noticeable cell swelling, and uneven flow restoration throughout the tissue [1]. Ischemia-reperfusion injury leads to significant renal function impairment, exacerbates histopathological damage, and increases apoptosis. These effects are primarily attributed to the excessive production of oxygen-free radicals and a reduction in antioxidant substances [2]. Renal tissue damage is driven primarily not by ischemia itself but rather by reperfusion, which occurs after an ischemic event is resolved and blood flow is restored, and it has a much more destructive effect than ischemia [3]. Renal IRI is caused by a cascade of events, including hemodynamic changes, oxidative stress, inflammation, apoptosis, and necrosis [4]. However, the specific contribution of each of these factors to the decline in glomerular filtration rate (GFR) remains unclear [5]. IRI involving renal tissues plays a role in the development of a clinical syndrome characterized by rapid renal dysfunction and elevated mortality rates. This condition is known as acute kidney injury (AKI). AKI affects about 15% of hospitalized adults and around a quarter of hospitalized children. More than half of the patients in intensive care have a higher incidence of AKI. Numerous studies showed a link between AKI and a higher risk of developing chronic kidney disease (CKD) [6]. Kidney injury after ischemia and reperfusion is mediated by proinflammatory mediators, such as cytokines, produced by tubular epithelial cells (TECs) in a rapid response to hypoxia during the ischemic period. During reperfusion, mitochondrial oxidative stress and reactive oxygen species (ROS) are responsible not only for DNA damage but also for further tissue injury. In addition, it results in inflammatory cell activation, interleukin secretion, and tumor necrosis factor-alpha (TNF-α) release. In turn, this increases tissue damage after reperfusion because it triggers an inflammatory response and encourages cell apoptosis [7]. The reperfused renal tissue exhibits an elevated accumulation of neutrophils and heightened levels of cytokines, including tumor necrosis factor-alpha (TNF-α) and interleukin-1 beta (IL-1β). The process of renal ischemia-reperfusion injury induces an excessive production of reactive oxygen species (ROS). These ROS contribute to modifications in membrane structure and function, causing cellular damage through processes such as lipid peroxidation in cell membranes involving species like superoxide radical (•O), hydrogen peroxide (H2O2), and hydroxyl radical (•OH). These changes in the kidney act as triggers for kidney injury and inflammation. The antioxidant defense system is a complex network that consists of several antioxidant enzymes, including superoxide dismutase (SOD), catalase (CAT), and glutathione peroxidase [8]. TAC detects the cumulative effect of total antioxidants found in blood and body fluids. During renal IRI, a decline in kidney tissue TAC levels was reported [9]. According to recent research, apoptosis of tubular cells has a major contribution to renal IRI pathophysiology and determines the severity of renal damage. In addition, studies proved that apoptosis following renal IRI induces inflammation [10]. Among the numerous caspase proteins, caspase-3 has been extensively studied, and it has been hypothesized that it is a key contributor to renal dysfunction [11]. Nuclear factor erythroid 2-related factor 2 (NrF2) is essential in cellular defense mechanisms, which are activated in response to upregulated oxidative stress [12]. There is evidence that chronic kidney disease causes an obvious downregulation in nuclear NrF2 expression and enhanced production of ROS [13]. Among enzymes activated by NrF2, heme oxygenase-1 (HO-1) is a promising therapeutic target for ischemia-reperfusion injury (IRI) due to its abundant antioxidant response elements (AREs) in its promoter region. HO-1 possesses potent endogenous antioxidant, anti-inflammatory, and anti-apoptotic activities. Its distribution among various tissues is wide, along with its protective effect on ischemia-reperfusion such as heart, lung, and brain [14, 15]. RAL hydrochloride (RAL), the initial FDA-approved selective estrogen receptor modulator (SERM) for postmenopausal osteoporosis prevention and treatment, later gained approval for breast cancer risk reduction and management [16]. RAL holds therapeutic promise in addressing obesity, dyslipidemia, and endothelial impairment. Nevertheless, its antioxidant and anti-inflammatory effects involving the cytoprotective HO system cannot be overlooked. SERMs exert their activity as agonists or antagonists in a tissue-specific manner [17]. RAL is effective in breast and endometrial cancer prevention due to its antagonistic effect on the breast and uterus. Utilizing its agonist properties in bones, RAL has been utilized for decades in the prevention and management of postmenopausal osteoporosis [18]. RAL may possess some benefits for kidney disease treatment [19]. The purpose of this study was to investigate the potential renoprotective effects of RAL on bilateral renal IRI in rats by investigating kidney function biomarkers, including urea and creatinine (U&Cr), inflammatory cytokines TNFα and IL-1β, the antioxidant marker total antioxidant capacity (TAC), pro-apoptotic marker caspase-3, and histopathological assessments.

## MATERIALS AND METHODS

### Animal preparation, treatment, and euthanasia

For this study, adult Sprague-Dawley rats aged 20-24 weeks and weighing between 200 to 350 grams were used. These rats were obtained from the Center of Control and Pharmaceutical Research, Ministry of Health, Baghdad, and were housed in the Animal Resources Centre at the College of Sciences, University of Kufa. The animal housing facility maintained controlled conditions, including a temperature of 24±2°C and humidity ranging from 60% to 65%, following a 12-hour light and 12-hour dark cycle. All rats were provided with a standard diet and access to tap water.

### Study design

The study employed an experimental design, with rats randomly assigned to four groups, each consisting of five rats, as follows:

1. Sham group: Rats underwent median laparotomy for approximately 2 hours and 30 minutes without ischemia induction.
2. Control group: Rats experienced bilateral renal ischemia for 30 minutes, followed by reperfusion with restored renal blood flow for 2 hours [20].
3. Vehicle group: Rats received an intraperitoneal injection of a mixture containing corn oil and DMSO (Medchemexpress/USA) 30 minutes before ischemia induction. Subsequently, they underwent 30 minutes of bilateral renal ischemia and 2 hours of reperfusion [21]. The vehicle group was established to mitigate any impact of DMSO and corn oil on the outcomes.
4. RAL treated group: Rats were administered an intraperitoneal injection of RAL 10 mg/kg [22] 30 min before ischemia induction, followed by 30 min bilateral renal ischemia and 2 hours of reperfusion [23].

### Experimental study model

At the beginning of the experiment, rats were weighed, and anesthesia was induced intraperitoneally using a combination of ketamine (100 mg/kg) and xylazine (10 mg/kg) [20]. To ensure surgical stability, the rats were placed on their backs and sedated for 5-10 minutes. Limbs and tails were secured with stickers. Hair was removed from the chest and abdomen by shaving, and 10% iodine spray was used to disinfect the skin. To make sure the rats were sufficiently anesthetized, the reflexes were tested by pinching the tail. The intestine was retracted, and the abdomen and both renal pedicles were exposed through a midline laparotomy incision. For the bilateral ischemia model, the renal pedicles were isolated by placing non-trauma microvascular clamps around the renal arteries and veins [21]. After a few minutes, the kidney's color changed from red to dark purple, accompanied by pale stains on the kidney surface, indicating blood flow blockage. The clamping was maintained for 30 minutes while the abdomen was covered with saline-moistened gauze to maintain rat hydration. After 30 minutes, the clamps were removed from the pedicles, allowing the restoration of renal blood flow, and the deep purple color of the kidneys turned pale red, which denotes the start of the two-hour reperfusion phase [22]. The abdominal cavity incision was sutured in two layers using three interrupted sutures after the kidney was placed back in its original position. Then, euthanasia was performed by deep anesthesia with ketamine and xylazine. Finally, blood and tissue samples were collected for analysis. The study's objective was to induce ischemia for 30 minutes, as research indicates kidney damage results from ischemia exceeding 20 minutes. Reversible damage is possible with ischemia lasting 20 to 40 minutes, while prolonged ischemia beyond 40 minutes may lead to permanent damage. The selected 30-minute ischemia period in this study aimed to ensure reversible damage without permanence [23].

### Preparation of RAL

Pure RAL hydrochloride powder was purchased from Med Chem Express Company, USA. Molecular Formula: C_28_H_27_NO_4_S.HCl. Chemical Name [6-hydroxy-2-(4-hydroxyphenyl)-benzothiophene-3-yl]-[4-[2-(1-piperidyl)ethoxy]phenyl]-methanon hydrochloride Cas number 82640-04-8. Purity ≥98% (HPLC). This compound is characterized by solubility exceeding 2.5 mg/mL (4.90 mM). For preparation, each solvent was sequentially added following the manufacturer's guidelines: 10% DMSO and 90% corn oil, as outlined in the instructions leaflet provided by the manufacturing company. This solution was prepared immediately prior to use, adhering to the Med Chem Express company's instructions. The administered drug dose was 10 mg/kg of rat weight, delivered intraperitoneally [24].

### Preparation of blood samples for determining kidney function parameters

At the end of the surgical procedure, following 2 hours of reperfusion, approximately 3.5-5 ml of blood was directly drawn from the heart of each rat. The collected blood samples were then placed in pre-labeled gel tubes without the addition of anticoagulant and allowed to incubate at 37°C for 30 minutes. Subsequently, each gel tube underwent centrifugation at 3000 rpm for 10 minutes to separate the serum. This serum was essential for assessing urea and creatinine levels, which were determined using an enzyme-linked immunosorbent assay (ELISA) kit [25].

### Tissue preparation to determine TNF-α, IL1-β, caspase-3, and TAC

At the end of the reperfusion period, the left kidney was removed and rinsed with an ice-cold isotonic solution of 0.9% to eliminate any blood clots before being divided into two parts. One part underwent homogenization using a high-intensity ultrasonic liquid processor with a ratio of 1:10 (w/v) in phosphate-buffered saline containing 1% Triton X-100 and a protease inhibitor cocktail [26]. The resulting homogenate was centrifuged at 14000 rpm for 20 minutes at a temperature of 4°C. Subsequently, the supernatant was collected to assess TNF-α, IL-1β, and caspase-3 using the ELISA technique and TAC using a colorimetric method.

For the ELISA measurements (TNF-α, IL-1β, and caspase-3):

- Reagents, standard solutions, and samples were prepared according to the provided instructions.
- The appropriate number of strips was determined and placed into frames for usage.
- 50 µl of the standard solution was added to the designated standard well.
- In sample wells, 40 µl of the sample was combined with 10 µl of anti-TNF-α, anti-IL-1β, or anti-caspase-3 antibody. Subsequently, 50 µl of streptavidin-HRP was added to both sample and standard wells. The plate was then incubated for 60 minutes at 37°C.
- The plate was gently washed five times with a wash buffer.
- 50 µl of substrate solution A was added to each well, followed by 50 µl of substrate solution B. The plate was incubated for 10 minutes at 37°C in darkness.
- Finally, 50 µl of stop solution was introduced to each well, causing the blue color to transition to yellow. The optical density (OD value) was measured at 450 nm within a 10-minute window.

### Tissue sampling for histopathology analysis and score damage grading

The remaining part of the left renal tissue underwent fixation in 10% formalin, then dehydrated in a series of alcohols, cleared in xylene, and finally embedded in paraffin to form the paraffin block. The tissue slide sections were cut into 5-mm-thick horizontal sections and stained with hematoxylin and eosin for histological analysis. Following fixation, a researcher blinded to the experimental treatment groups carried out a score evaluation. Light microscopy was used to grade tissue sections for degeneration and necrosis using quantitative measurements and a scoring system [27]. Tubular epithelial swelling, brush border loss, vacuolar degeneration, and cast formation are signs of renal tubule damage [28]. The degree of magnification that estimated kidney injury was X400. In the section of kidney tissue, the score of histological changes was determined as a renal tubular damage percentage as follows:

Score 0 represents normal

Score 1 represents <25% of damaged tubules

Score 2 represents 26%-50% of damaged tubules

Score 3 represents 51% -75% of damaged tubules

Score 4 represents 76% -100% of damaged tubules [29].

### Statistical analysis

Statistical analysis of the experimental results was conducted using GraphPad Prism version 7. Tukey's multiple comparisons and one-way analysis of variance (ANOVA) were employed to investigate the significance of differences between the various groups. The data were expressed as mean ± standard error of the mean (SEM), and a p-value of less than 0.01 was considered statistically significant.

## RESULTS

### Effects of IRI and RAL on kidney function parameters (blood urea nitrogen and serum creatinine)

**Figure 1:**
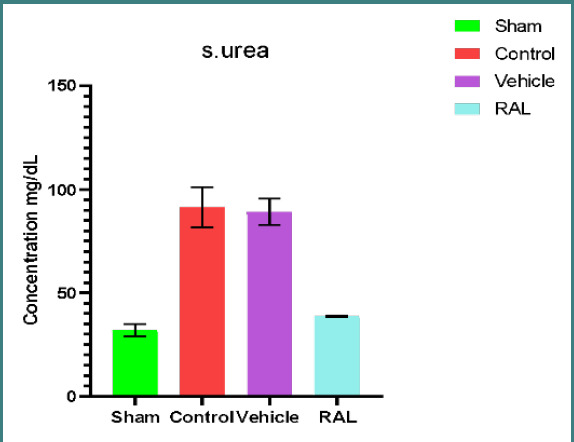
Mean serum urea (mg/dl) levels across all groups. Data presented as mean ± SEM, N=5. Statistical analysis was conducted using one-way ANOVA followed by Tukey multiple comparisons. Comparison of control and vehicle groups with the sham group: p-value≤0.01; comparison of RAL group with control & vehicle groups: p-value≤0.01; RAL: Raloxifene.

**Figure 2:**
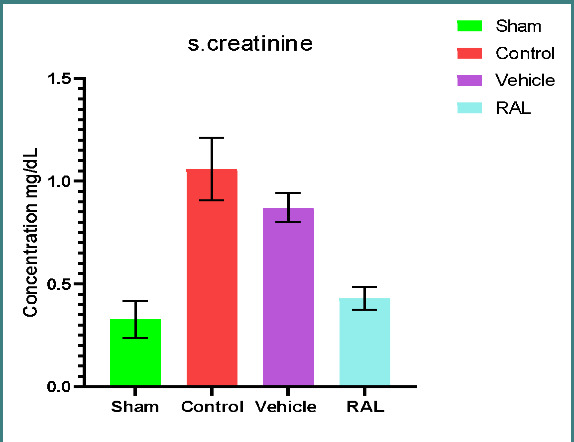
Mean serum creatinine (mg/dl) levels across groups. The data are expressed as mean ± SEM, N=5. A one-way ANOVA followed by Tukey multiple comparisons was utilized for statistical analysis. Control & vehicle groups vs. sham group: p-value≤0.01; RAL group vs. control & vehicle groups: p-value≤0.01; RAL: Raloxifene.

There was a significant increase in serum urea (U) and creatinine (Cr) levels in both control and vehicle groups compared to the sham group (p≤0.01). However, no significant difference was observed between the control and vehicle groups (p≥0.01). The RAL-treated group had a significant decrease in serum urea and creatinine levels compared to the control and vehicle groups (p≤0.01). Mean serum U and Cr levels are shown in Figures 1 and 2, respectively.

### Effects of IRI and RAL on inflammatory markers TNF-α and IL1-β

**Figure 3:**
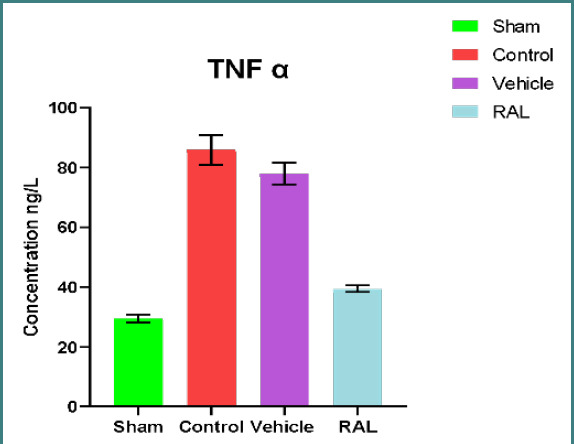
Mean renal tissue of TNF-α (ng/l) levels across groups at the end of the study. Data are expressed as mean ± SEM, N=5. ANOVA followed by Tukey multiple comparisons was utilized for statistical analysis. Control & vehicle groups vs. sham group: p-value≤0.01; RAL group vs. control & vehicle groups: p-value≤0.01; RAL: Raloxifene.

**Figure 4:**
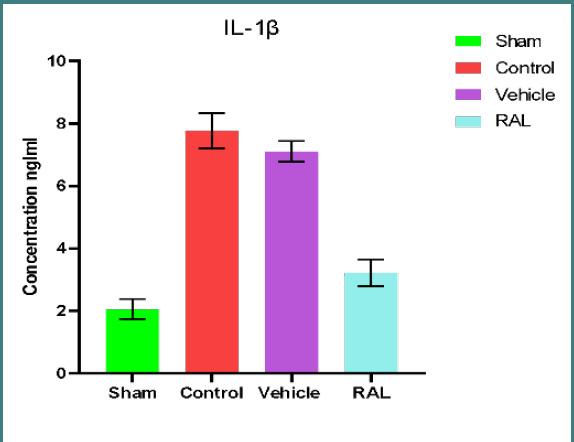
Mean serum IL-1β (ng/ml) levels across groups. Data are expressed as mean ± SEM, N=5. ANOVA followed by Tukey multiple comparisons was utilized for statistical analysis. Control & vehicle groups vs. sham group: p-value≤0.01; RAL group vs. control & vehicle groups: p-value≤0.01; RAL: Raloxifene.

There was a significant (p≤0.01) increase in renal tissue levels of TNF-α and IL-1β in the control and vehicle groups compared to the sham group. However, no significant difference (p≥0.01) was found between the control and vehicle groups. The RAL-treated group showed a significant (p≤0.01) decrease in TNF-α and IL-1β levels compared to control and vehicle groups. The mean levels of TNF-α and IL-1β are shown in Figures 3 and 4, respectively.

### Effects of IRI and RAL on caspase 3

**Figure 5:**
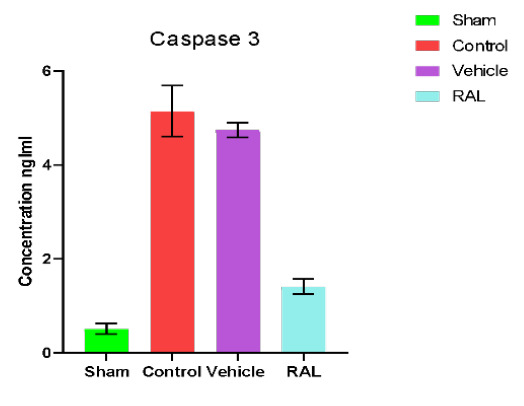
Mean serum caspase-3 (ng/ml) levels across groups. The data are expressed as mean ± SEM, N=5. ANOVA followed by Tukey multiple comparisons was utilized for statistical analysis. Control & vehicle groups vs. sham group: p-value≤0.01; RAL group vs. control & vehicle groups: p-value≤0.01; RAL: Raloxifene.

There was a significant (p≤0.01) increase in the renal tissue levels of caspase 3 in the control and vehicle groups compared to the sham group. However, no significant difference (p≥0.01) was found upon comparing the control and vehicle groups. Conversely, the RAL-treated group exhibited a significant (p≤0.01) reduction in renal tissue caspase 3 levels compared to the control and vehicle groups. Mean renal tissue caspase 3 levels are shown in Figure 5.

### Effects of IRI and RAL on TAC

**Figure 6:**
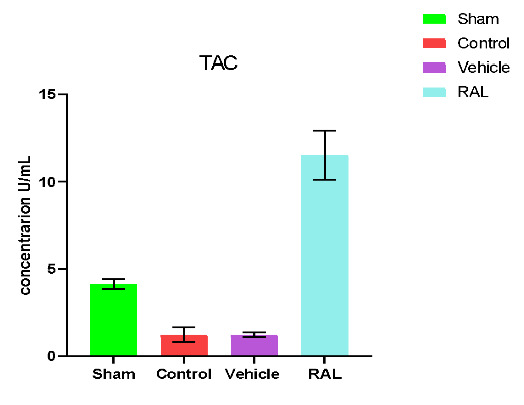
Mean serum TAC (U/ml) levels across groups. The data are expressed as mean ± SEM, N=5. ANOVA followed by Tukey multiple comparisons was utilized for statistical analysis. Control & vehicle groups vs. sham group: p-value≤0.01; RAL group vs. control & vehicle groups: p-value≤0.01; RAL: Raloxifene.

There was a significant (p≤0.01) decrease in the levels of TAC in the control and vehicle groups compared to the sham group. However, no significant difference (p≥0.01) was found when comparing the control and vehicle groups. Conversely, the RAL-treated group had a significant (p≤0.01) increase in renal tissue TAC levels compared to the control and vehicle groups. Mean renal tissue TAC levels are shown in Figure 6.

### Histopathological examination

**Figure 7:**
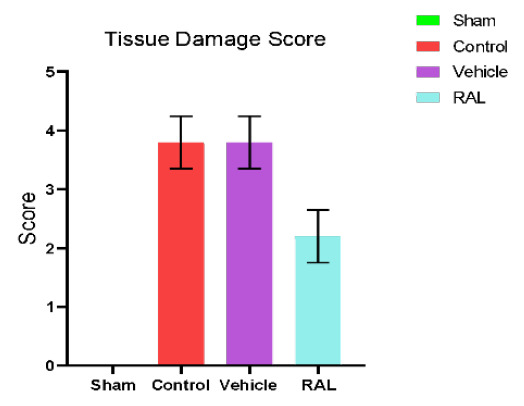
Mean severity score of histopathological kidney tissue across groups. The data are expressed as mean ± SEM, N=5. ANOVA followed by Tukey multiple comparisons was utilized for statistical analysis. Control & vehicle groups vs. sham group: p-value≤0.01; RAL group vs. control & vehicle groups: p-value≤0.01; RAL: Raloxifene.

**Figure 8:**
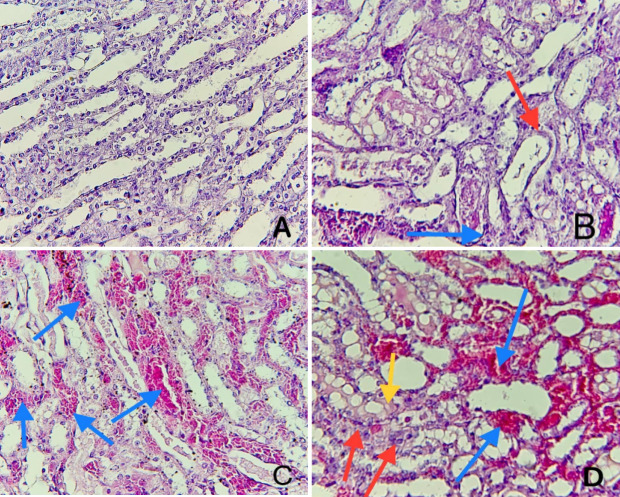
Hematoxylin and eosin staining of renal tissues. A. Sham group: Cross-section of the left kidney displaying normal histology. Magnification: 400×. B. Control group: Cellular swelling and cytoplasmic eosinophilia (red arrows), vacuolated epithelium, and Eosinophilic cast (blue arrow). Magnification: 400×. C. Vehicle group: Vascular congestion and hemorrhage (blue arrow). Magnification: 400×. D. RAL-treated group: Cross-section of the left kidney revealing a score of 2. Renal ischemic changes affecting 35% of renal tubules, including cellular swelling and cytoplasmic eosinophilia (red arrows), vascular congestion, and hemorrhage (blue arrow), as well as cytoplasmic vacuoles (yellow arrow). Magnification: 400×.

A histopathological examination was conducted at the end of the study, with the damage score and histological findings presented in Figures 7 and 8. The cross-section of kidney tissue from the sham group had a normal renal structure, normal glomerulus, and normal kidney tubules, as shown in Figure 8A. On the other hand, the renal tissue cross-section of the control group displayed abnormal renal structure and severe kidney damage, affecting nearly 80% of renal tubules. This damage was characterized by increased eosinophilia, vacuolated epithelium, and the presence of Eosinophilic cast, resulting in a high severity score mean (severity score mean=4), as shown in Figure 8B. Similarly, the kidney tissue cross-section of the vehicle group exhibited compromised kidney structure and severe renal injury, with approximately 80% of renal tubules affected, including dilatation of renal tubules, loss of brush borders, increased cytoplasmic eosinophilia, vascular congestion, and hemorrhage (Figure 8C). In contrast, the kidney tissue cross-section of the RAL-treated group revealed mild to moderate changes in kidney structure, with damage affecting around 35% of renal tubules (Figure 8D).

## DISCUSSION

Renal ischemia-reperfusion injury (IRI) is characterized by a sudden decrease in blood supply to the kidney, followed by the restoration of blood flow containing oxygen. This sequence of events gives rise to a range of pathological issues, including damage to the glomerulus and tubules [30-32]. During ischemia-reperfusion injury (IRI), kidney tissue sustains damage due to the excessive accumulation of reactive oxygen species (ROS), leading to subsequent oxidative stress. When the blood flow is restored during the reperfusion period, it will generate further AKI involved in apoptosis and cell death. Renal IRI is commonly observed as a predictable complication in renal surgical procedures, including partial nephrectomy and renal transplantation. The present experimental research demonstrated a significant (p≤0.01) increase in urea and creatinine serum levels in the control and vehicle groups compared to the sham group after IRI. These findings are consistent with prior studies [33], which explained that the reduction in the supply of oxygen and nutrients results in kidney dysfunction and nitrogenous waste product accumulation like creatinine and urea in the body. Administering RAL prior to ischemia induction resulted in a reduction in serum levels of urea and creatinine. This outcome is consistent with findings from other studies [34], which can be attributed to the anti-inflammatory impact of RAL, manifested by a significant downregulation of TNF-α and other cytokines in renal parenchymal cells [35].

In this experimental study, both the control and vehicle groups showed severe renal injury, which was significantly (p≤0.01) greater than the sham group. These histological changes included cellular swelling, tubule dilation, cytoplasmic eosinophilia, disappearance of brush borders, cell desquamation, cast formation in tubular lumens, vascular congestion, interstitial inflammation, and hemorrhage. These results are consistent with findings from other studies, indicating that IRI can lead to the loss of brush boundaries, tubular dilatation, tubule casting, and vacuolization of epithelial cells [36]. Both the ischemia and reperfusion periods contribute to renal damage, inducing hypoxia and excessive AKI production. Another study demonstrated that bilateral IRI can cause severe damage to renal tubules, reduce the number of normal renal cells, enhance the expression of genes responsible for macrophage accumulation, and consequently lead to alterations in cell proliferation, leukocyte migration to the injury site, suppression of cellular immunity, and activation of inflammatory responses that further contribute to renal damage [37].

Our study revealed a significant decrease in kidney tissue damage severity following the administration of RAL prior to ischemia induction, as compared to tissue injury observed in both the control and vehicle groups (p≤0.01). The mean score severity in this pretreatment group showed mild to moderate renal injury, corresponding to other studies. One study showed significantly less renal interstitial fibrosis in the RAL-treated group than in controls. In another study, RAL reduced renal tubular damage in a mice model with hereditary glomerulonephritis [38]. This renoprotective effect in rats can be explained by the ability of RAL to alter the balance of antioxidants by reducing levels of oxidative stress and increasing redox system activity. This study showed that the level of TNFα and IL-1β in damaged kidney tissues after IRI was significantly higher in both the control and vehicle groups (p≤0.01) compared to the sham group, corresponding with other studies. In one study, rats were subjected to 30 minutes of ischemia followed by 2 hours of reperfusion and exhibited increased TNF-α levels due to upregulated cytokines or chemokines, along with activation of inflammatory cells that mediate injury. Another study highlighted that the level of IL-1β was remarkably increased in the injured kidney tissues following half an hour of ischemia and two hours of reperfusion in a rat model [37]. Moreover, one study involving renal IRI in rat models also revealed a significant elevation of IL-1β levels in kidney tissues after ischemia [39-41].

The inflammatory response plays a pivotal role in the development of renal IRI. This process involves the notable accumulation of neutrophils and the production of inflammatory factors, chemokines, and adhesion molecules to further activate neutrophils. This activation results in chemotaxis, causing neutrophils to infiltrate and aggregate, subsequently exacerbating renal injury. Administering RAL prior to inducing ischemia resulted in a significant reduction (p≤0.01) in the levels of inflammatory cytokines (TNF-α and IL-1β) in ischemic kidney tissues compared to their levels in both the control and vehicle groups. These findings suggest that RAL exhibits renoprotective activity in renal tissues subjected to IRI. Our results are in line with prior studies that demonstrated the ability of RAL to suppress the production of inflammatory mediators TNF-α and IL-1β. These outcomes can be attributed to the effect of RAL on upregulating HO-1 gene expression, which exerts anti-inflammatory, antioxidant, and anti-apoptotic effects. HO-1 overexpression is known to lead to the downregulation of inflammatory molecule expression [42-43].

The expression of HO-1 is regulated by multiple pathways, including the Nrf2 pathway, which is activated by RAL, which also has the ability to produce anti-inflammatory activity and further reduce tissue levels of inflammatory mediators [44]. Our study showed a significant (p≤0.01) decrease in TAC levels in renal tissues in both control and vehicle groups following IRI compared to the sham groups. This decline in TAC levels can be attributed to the elevated oxidative stress and formation of reactive oxygen species (ROS) in the injured kidney tissues of the control and vehicle groups in contrast to the normal tissues of the sham group. This finding aligns with prior research, where studies have demonstrated the pivotal role of oxidative stress in the pathogenesis of kidney IRI. One study highlighted a significant decline in TAC levels following IRI induction. Another study explained the significant decrease in TAC levels following IRI, mainly due to the reoxygenation of the ischemic kidney, which triggered ROS formation and subsequently activated cytokines and chemokines.

The antioxidant defense system degrades after IRI, and the amount of MDA, a byproduct of lipid peroxidation, increases while decreasing antioxidant levels. These procedures all cause cell apoptosis. In addition, during renal IRI, the ischemic kidney tissue undergoes excessive free radical production, further exacerbated during the reperfusion phase, thereby intensifying oxidative stress and leading to oxidative damage, apoptosis, and cellular death [45].

This study found that the administration of RAL prior to the induction of ischemia significantly (p≤0.01) increased the levels of total antioxidant capacity (TAC) while reducing oxidative stress and the production of free radicals within ischemic renal tissues when compared to the control and vehicle groups. These findings suggest that RAL has an antioxidant effect on damaged kidney tissues that have experienced ischemia and reperfusion. This conclusion is consistent with previous research findings. One research explained that RAL significantly increased TAC levels after IRI induction. This may be due to the effectiveness of RAL as an antioxidant agent to reduce plasma oxidative stress. Another study demonstrated the antioxidant effect of RAL by increasing a signaling pathway involved in defending against oxidative stress, Nrf2/HO-1. This signaling pathway has been identified as a crucial component of the antioxidant defense system in humans, enabling cells to bolster their resistance against oxidative stress, enhance the expression of antioxidant enzymes, maintain redox balance, and ultimately reduce ROS-induced cellular damage [46].

Renal tissue levels of caspase 3 in both control and vehicle groups were significantly elevated compared to the sham group after IRI (p≤0.01). This observation is in line with findings from other studies. According to an experimental study involving bilateral renal ischemia for 60 min followed by 24 hrs. reperfusion in a rat model, caspase 3 level in the sham group was lower than in the control group. An experimental study design using a rat model showed that caspase 3 levels significantly increased in both the control and vehicle groups compared to the sham group after 30 min of ischemia and 2 hours of reperfusion [47]. The detrimental effects of ischemia-reperfusion injury on cellular health are mediated by the formation of ROS during the injury phase, which subsequently triggers an inflammatory response and contributes to the development of acute kidney injury. In addition, the production of ROS triggers the signaling pathways that cause apoptosis and necrosis in cells. The present research revealed that administering RAL prior to ischemia-reperfusion could significantly (p≤0.01) downregulate caspase 3 expression in ischemic kidney tissues compared to the control and vehicle groups. These results align with the outcomes of other studies. In one experiment, treatment with RAL led to a significant reduction in caspase 3 protein expression (p<0.05) [48]. Furthermore, additional research demonstrated that RAL treatment substantially reduced the percentage of apoptotic cells to 0.009% (p≤0.05) by decreasing caspase 3 protein levels.

This study suggests that RAL inhibits oxidative stress-induced apoptosis in endothelial cells through a mechanism linked to the antioxidant effect of selective estrogen receptor modulator (SERM). A recent study showed a link between oxidative stress and apoptosis. Recent investigations have established a connection between oxidative stress and apoptosis, where excessive ROS production at ischemic sites disrupts mitochondrial ATP synthesis, dissipates mitochondrial membrane potential, leads to the opening of mitochondrial permeability transition pores, releases cytochrome C, and ultimately activates the caspase amplification cascade, culminating in cellular apoptosis [49]. RAL has demonstrated its ability to enhance the expression of the HO-1 gene, contributing to its antioxidant mechanism that subsequently reduces caspase cascade and apoptosis [50, 51].

## CONCLUSION

RAL had a significant renoprotective effect on renal IRI. These effects were evidenced by its ability to enhance kidney function parameters (urea and creatinine), suppress the expression of inflammatory mediators (TNFα and IL-1β) – thereby affirming its anti-inflammatory action – and enhance cellular antioxidant capacity (TAC) to counteract the presence of free radicals and ROS in ischemic kidney tissues, underscoring its antioxidant activity. Furthermore, RAL demonstrated its potential as an anti-apoptotic agent by reducing the pro-apoptotic marker caspase 3 levels in renal tissue.
